# Association of GLP-1 Receptor Agonist Use with Hypersomnolence: A Real-world Cohort Analysis

**DOI:** 10.1007/s40200-026-01929-0

**Published:** 2026-03-18

**Authors:** Louie Kamel-Abusalha, Ahmed M. Afifi, Abdallatif Dawoud, Zumair Hayath, Muhammad Fouad Bouso, Andre Aguillon, Ragheb Assaly

**Affiliations:** 1 University of Toledo Medical Center, Toledo, OH USA; 2 Department of Medicine, New York-Presbyterian Brooklyn Methodist Hospital, Brooklyn, NY USA; 3 Division of Pulmonary, Critical Care and Sleep Medicine, Medical Intensive Care Unit, 3000 Arlington Ave, Toledo, OH 43614 USA; 4 3000 Arlington Ave, Toledo, Research Associate, OH 43614 USA

**Keywords:** Somnolence, Sleepiness, Glucagon like peptide-1, parasomnia, Restless legs syndrome, Iron deficiency anemia

## Abstract

Our study sought to evaluate the association between Glucagon-like peptide-1 receptor agonist use and the development of hypersomnolence at 1 and 5-year timepoints. A retrospective cohort study was done using TriNetX’s research network. Patients aged 18–50 with type-2 diabetes mellitus or obesity who underwent polysomnography were included. Patients were excluded if they had any prior use of antidepressants. Patients who used Glucagon-like peptide-1 receptor agonists were 1:1 propensity matched to patients without Glucagon-like peptide-1 receptor agonist exposure. The primary outcome was new onset hypersomnolence with secondary outcomes including parasomnia, narcolepsy/cataplexy, disturbed sleep, restless legs syndrome, iron deficiency, and Parkinsonism. Hazard ratios and risk ratios were calculated at 1- and 5-years. Kaplan-Meier analyses and log-rank tests were performed for time to event comparisons. After matching, there were two equal cohorts of 118,993 patients. The Glucagon-like peptide-1 receptor agonist cohort was associated with increased hypersomnolence at 1-year (HR 1.21, 95% CI 1.10–1.32; *p* < 0.001) and 5-years (HR 1.23, 95% CI 1.14–1.32; *p* < 0.001). The absolute risk increase was approximately 0.4% at 1 year and 0.7% at 5 years. Iron deficiency was significantly increased at 5-years (HR 1.11, 95% CI 1.06–1.16; *p* < 0.001). A decreased hazard of Parkinsonism was observed at 5-years (HR 0.26, 95% CI 0.08–0.80; *p* = 0.012), although event counts were low. Time to event analyses did not demonstrate statistically significant associations for parasomnia, disturbed sleep, restless legs syndrome, or narcolepsy/cataplexy. These findings highlight the need for further studies to evaluate the effects of Glucagon-like peptide-1 receptor agonists on sleep regulation and the neurophysiological consequences of these agents.

**Supplementary Information:**

The online version contains supplementary material available at 10.1007/s40200-026-01929-0.

## Introduction

Glucagon-like peptide-1 receptor agonists (GLP-1RA) have become a cornerstone therapy in the treatment of type 2 diabetes mellitus (T2DM), offering glycemic control, weight reduction, and favorable cardiovascular profiles. More recently, agents such as semaglutide and liraglutide have received FDA approval for obesity treatment in individuals without diabetes, expanding on previous indications [1].

While the peripheral metabolic actions of GLP-1RAs are well established, accumulating evidence suggests that GLP-1RAs exert significant central nervous system (CNS) effects. GLP-1 receptors are widely expressed throughout the brain, including regions such as the hypothalamus and brainstem nuclei [2–4]. GLP-1 signaling has been shown to suppress ghrelin, a hunger promoting hormone that stimulates orexin producing neurons in the lateral hypothalamus [5]. Orexins have a critical role in maintaining arousal and stabilizing sleep wake transitions [6]. Preclinical studies suggest that GLP-1RAs may interact with this system by dampening orexinergic activity, potentially leading to hypersomnolence, altered REM dynamics, and other sleep-related disturbances [6, 7].

Despite these mechanistic insights, the effects of GLP-1RAs on human sleep physiology remain poorly understood. Little is known about how these agents are associated with hypersomnolence, parasomnia, or narcolepsy-like symptoms in the broader population [8]. Post-marketing pharmacovigilance analyses have identified neurological adverse event signals associated with GLP-1RA use, including reports of lethargy and central mediated symptoms [9]. This study aims to investigate the association between GLP-1 RA use and the onset of hypersomnolence, parasomnia, and related outcomes at 1- and 5-year timepoints, using a large real-world dataset of patients.

Materials & Methods.

### Ethical Approval

This retrospective study is exempt from informed consent. The data reviewed is a secondary analysis of existing data, does not involve intervention or interaction with human subjects, and is de-identified per the de-identification standard defined in Section § 164.514(a) of the HIPAA Privacy Rule. The process by which the data is de-identified is attested to through a formal determination by a qualified expert as defined in Section § 164.514(b)(1) of the HIPAA Privacy Rule. This formal determination by a qualified expert refreshed on December 2020.

The study protocol was reviewed and approved by the University of Toledo Institutional Review Board (IRB #302402-UT). All study procedures complied with institutional policies and federal regulations governing human subjects research. The IRB approved the protocol titled ‘Association of GLP-1 Receptor Agonist Use with Hypersomnolence: A Real-World Cohort Analysis’.

### Study Design

This was a retrospective cohort study utilizing the TriNetX Research Network in July 2025. TriNetX has data including information on diagnosis, medications, procedures, laboratory values, and genomic information on approximately 157 million patients from 107 healthcare organizations (HCO). Codes from diagnosis used International Classification of Disease (ICD-10-CM), laboratory tests used Logical Observation Identifiers Names and Codes (LOINC), TriNetX curated codes (TNX), and procedures via Current Procedural Terminology (CPT) codes. This study was reported in accordance with the STROBE (Strengthening the Reporting of Observational Studies in Epidemiology) statement guidelines for cohort studies [27] which is listed in the supplementary information.

### Cohorts

Within the TriNetX research network, patients who were aged 18–50 years and had a diagnosis of T2DM or obesity and underwent polysomnography were identified. Polysomnography was used to identify a population who was actively undergoing an evaluation for sleep-related symptoms. This method was implemented to enrich the cohort with documented sleep concerns, thereby increasing the likelihood of outcome capture in administrative data. Polysomnography results themselves were not used to define outcomes; therefore, this requirement should only be interpreted as a cohort selection strategy rather than confirmation of sleep disorder diagnosis.

The study was restricted to patients who had no history of antidepressant use to reduce any confounding by centrally acting agents.

**Fig. 1:**
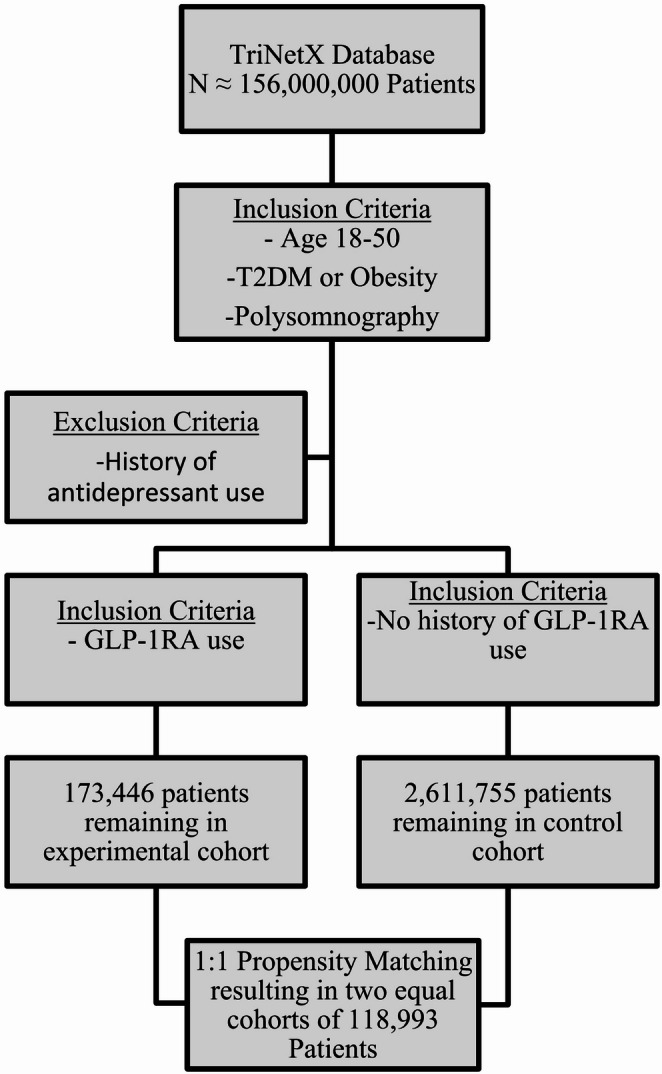
Study selection flow diagram

Two cohorts were constructed using the criteria listed. The experimental cohort included patients who used GLP-1 receptor agonists (GLP-1RA) while the control group did not have any previous use of these agents. A total of 79 HCOs responded with patient data meeting the study criteria at the time of query. The cohorts were 1:1 propensity matched based on demographics, preexisting conditions, and medication use all listed in Table 1. Included in the propensity matching were medications and conditions that could introduce confounding bias such as obstructive sleep apnea, mood disorders, anxiety disorders, chronic pain syndromes, benzodiazepines, and antihistamines. A patient flow diagram shows the cohort selection process in Fig. 1.

**Table 1:** Characteristics in unmatched population and propensity matched population

Characteristic	Unmatched GLP-1 (*n* = 173,466)	Unmatched Control (*n* = 2,611,755)	SMD Unmatched	Matched GLP-1 (*n* = 118,993)	Matched Control (*n* = 118,993)	SMD Matched
Age (mean ± SD)	39.9 ± 8.1	36.0 ± 9.3	0.452	39.3 ± 8.1	39.7 ± 8.0	0.049
Age at Index (mean ± SD)	37.4 ± 8.1	30.1 ± 10.4	0.778	36.6 ± 8.1	37.2 ± 8.0	0.064
Female (%)	61.5	60.1	0.029	62.9	61.4	0.031
Male (%)	38.5	39.9	0.029	37.1	38.6	0.031
Black (%)	25	21.3	0.089	23.3	22.3	0.023
White (%)	51.8	52.4	0.012	53.7	54.7	0.02
Asian (%)	5.8	4.3	0.07	5.2	5.1	0.004
American Indian/Alaska Native (%)	0.7	0.7	0.005	0.7	0.8	0.006
Pacific Islander (%)	1.4	1.3	0.01	1	0.8	0.025
Other Race (%)	5.9	7.1	0.048	6	6.1	0.004
Unknown Race (%)	9.4	13	0.116	10	10.2	0.005
Hispanic or Latino (%)	15.3	17.8	0.068	15.4	15.9	0.014
Not Hispanic or Latino (%)	63.4	56.1	0.149	61.3	59.5	0.037
Unknown Ethnicity (%)	21.4	26.1	0.112	23.3	24.6	0.031
Obesity (%)	60.9	7.4	1.366	49.9	49.5	0.008
Diabetes Mellitus (%)	41.8	4.5	0.985	26.4	27	0.014
Heart Failure (%)	2.6	0.7	0.153	2	2	0.002
CKD (%)	3.2	1	0.155	2.5	2.3	0.012
Sleep Apnea (%)	16.5	3.3	0.453	10.9	9.8	0.036
Hyperlipidemia (%)	21.8	3.4	0.578	13.9	12.7	0.034
Mood Disorders (%)	9.7	4.4	0.207	8.4	8.1	0.011
Anxiety Disorders (%)	16	6.6	0.302	13.8	13.1	0.019
Chronic Pain Syndrome (%)	0.5	0.2	0.058	0.4	0.4	< 0.001
Metformin Use (%)	37.1	2.7	0.956	21.2	20.6	0.015
Antihypertensives (%)	7.6	2.7	0.221	6.7	6.8	0.004
SGLT2 Inhibitors (%)	7.9	0.2	0.396	3.4	3.1	0.017
DPP-4 Inhibitors (%)	5.1	0.1	0.313	2.1	1.8	0.02
Benzodiazepines (%)	23.6	10.2	0.364	19.9	18.1	0.046
Antihistamines (%)	34.8	18.1	0.385	29.6	26.5	0.069

### Outcomes

Patients with any prior history of an outcome of interest were excluded from each outcome-specific analysis to ensure capture of incident events. The primary outcome for this study was new onset hypersomnolence, defined by ICD-10 codes hypersomnia (G47.1) and somnolence (R40.0). Secondary outcomes included parasomnia (G47.5), narcolepsy/cataplexy (G47.4), disturbed sleep including insomnia and REM behavior disorder (G47.0, G47.52, G47.6, G47.8, G47.9), restless legs syndrome (G25.81), iron deficiency defined by low ferritin levels (LOINC: 2276-4, < 75 ng/mL), and Parkinsonism (G20).

### Statistical Analysis

TriNetX’s greedy nearest neighbor matching algorithm was used for the 1:1 propensity scoring matching to control for any differences between the cohorts. Propensity scoring used demographic characteristics, baseline comorbidities, and medication use listed in Table 1. A standardized mean difference of < 0.10 indicated an acceptable match for covariate balancing.

Risk ratios were calculated with their corresponding 95% confidence intervals (CI) to assess the relative risk at each outcome timepoint at 1 and 5-years. Kaplan-Meier survival curves were generated to assess timet o event data with log rank tests to evaluate group differences. Hazard ratios were also estimated using Cox-proportional hazard models. A two tailed p*-*value of < 0.05 indicated a statistically significant result.

We created forest plots using R version 4.5.1 RStudio with ggplot2 package to display the RR and HR estimates and 95% CI for each outcome at 1-year and 5-year time points. Each point represents the RR, and horizontal lines represent 95% CI with a reference line at RR/HR = 1 [28–31].

**Fig. 2:**
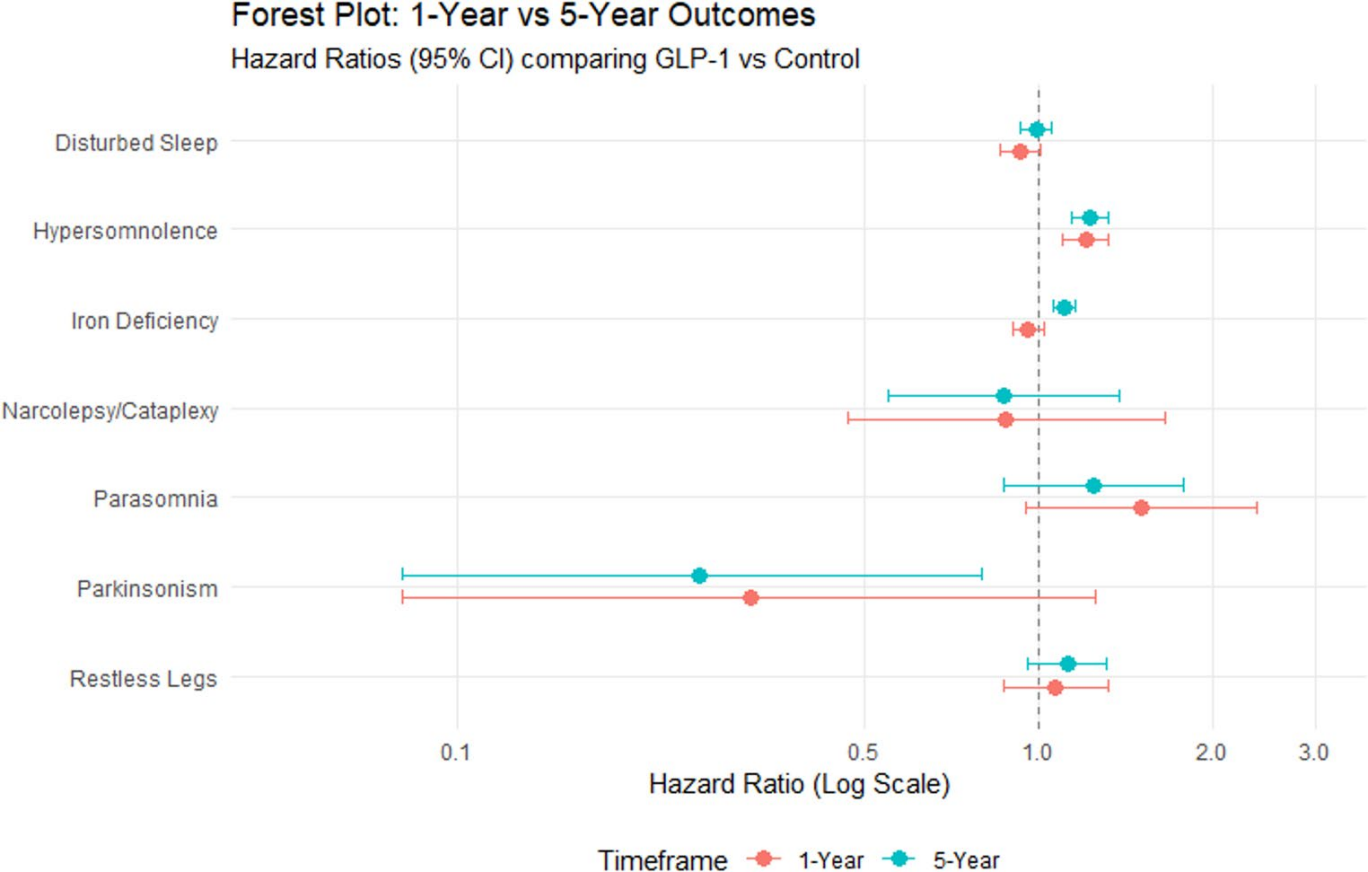
Forest plot of 1- and 5-year hazard ratio values grouped by outcome

## Results

After application of the inclusion and exclusion criteria, 173,446 individuals were identified for the experimental group (GLP-1 RA users) and 2,611,755 for the control group. After 1:1 propensity matching, there were two equal cohorts of 118,993 patients for use in the outcome analysis. Baseline characteristics were well balanced with standardized mean differences < 0.10 across all covariates. The mean age was approximately 39 years old for both cohorts and ~ 62% female population. Demographic data and baseline characteristics for the population studied are listed in Table 1.

### Primary Outcome: Hypersomnolence

Our Primary outcome examined was incident hypersomnolence. In time to event analysis, GLP-1RA use was associated with significantly increased hazard of hypersomnolence at both the 1-year and 5-year time points. At 1-year the HR was 1.21 (95% CI, 1.10–1.32; *p* < 0.001) with this association persisting at 5-years (HR 1.23, 95% CI, 1.14–1.32; *p* < 0.001). RR was directional consistent with these findings, with a RR of 1.61 (95% CI, 1.46–1.76) at 1-year and 1.72 (95% CI, 1.60–1.85) at 5-years.

### Secondary Outcomes

#### Parasomnia

At both timepoints, parasomnia did not reach statistical significance in time to event analysis. At 1-year the HR was 1.50 (95% CI, 0.95–2.38; log-rank *p* = 0.083) and at 5-years the association remained non-significant (HR 1.24, 95% CI, 0.87–1.78; *p* = 0.234). While cumulative incidence analysis demonstrated elevated RR (1-year RR 2.00; 5-year RR 1.75), these findings were not supported by the time to event analysis.

#### Narcolepsy/Cataplexy

No statistical association was observed between GLP-1RA use and narcolepsy/cataplexy in time to event analysis. At 1-year the HR was 0.88 (95% CI, 0.47–1.65; *p* = 0.682). At 5-years, the HR remained non-significant (HR 0.87, 95% CI, 0.55–1.38; *p* = 0.561). RR estimates were consistent with these findings with RRs of 1.17 at 1-year and 1.21 at 5-years. Overall counts were low and confidence intervals were wide, limiting precision of these estimates.

#### Disturbed Sleep

Disturbed sleep demonstrated a non-significant HR at 1-year (HR 0.93, 95% CI, 0.86–1.01; *p* = 0.095) and at 5-years (HR 0.99, 95% CI, 0.93–1.05; *p* = 0.750). The RRs were modestly elevated (1-year RR 1.25; 5-year RR 1.40), but the time to event analysis did not demonstrate a statistically significant association.

#### Restless Legs Syndrome

The HR for RLS was 1.07 (95% CI, 0.87–1.32; *p* = 0.509) at 1-year and 1.12 (95% CI, 0.96–1.31; *p* = 0.157) at 5-years, neither of which reached statistical significance. RR were elevated (1-year RR 1.44; 5-year RR 1.59), but these cumulative incidence differences were not confirmed in survival analysis.

#### Iron Deficiency

Iron deficiency demonstrated no significant association at 1-year in time to event analysis (HR 0.96, 95% CI, 0.90–1.02; *p* = 0.142). However, at 5-years, GLP-1RA use was associated with a significantly increased hazard of iron deficiency (HR 1.11, 95% CI, 1.06–1.16; *p* < 0.001). RR were consistent with this pattern (1-year RR 1.26; 5-year RR 1.55), with statistical significance observed at 5-years.

#### Parkinsonism

No significant difference was observed at 1-year (HR 0.32, 95% CI, 0.08–1.25; *p* = 0.085). At 5-years a statistically significant decreased hazard of parkinsonism was observed in the GLP-1RA cohort (HR 0.26, 95% CI, 0.08–0.80; *p* = 0.012), although the absolute event numbers were low. RR estimates were consistent with these findings at 5-years (RR 0.91), though interpretation should be cautious given the small number of events.

#### Interpretation of HR and RR Measures

Because this study utilized time to event methodology with censoring, HR derived from Coxsure proportional hazard models represent the primary inferential measure. RR reflects cumulative incidence at fixed time points and does not account for differential follow-up time. Discrepancies between HR and RR may occur when event timing differs between groups. For this reason, interpretation in this study prioritizes HR estimates over RR estimates.

Outcomes are listed in Tables 2 and 3 with a forest plot representation of HRs in Fig. 2.

**Tables 2:** 1-year outcomes after matching

Outcome	GLP-1 Cohort, *n* (%)	Control Cohort, *n* (%)	Risk Difference (%)	RR	HR (95% CI)	Log-rank *p*-value
Hypersomnolence	1,165 (1.0)	739 (0.6)	0.4	1.61	1.21 (1.10–1.32)	< 0.001
Parasomnia	54 (0.05)	27 (0.02)	0.03	2	1.50 (0.95–2.38)	0.083
Narcolepsy/Cataplexy	21 (0.02)	18 (0.02)	0	1.17	0.88 (0.47–1.65)	0.682
Disturbed Sleep	1,314 (1.2)	1,070 (0.9)	0.3	1.25	0.93 (0.86–1.01)	0.095
Restless Legs	221 (0.2)	154 (0.1)	0.1	1.44	1.07 (0.87–1.32)	0.509
Iron Deficiency	2,316 (2.1)	1,946 (1.7)	0.4	1.26	0.96 (0.90–1.02)	0.142
Parkinsonism	10 (0.01)	10 (0.01)	0	1	0.32 (0.08–1.25)	0.085

**Tables 3:** 5-year outcomes after matching

Outcome	GLP-1 Cohort, *n* (%)	Control Cohort, *n* (%)	Risk Difference (%)	RR	HR (95% CI)	Log-rank *p*-value
Hypersomnolence	2,015 (1.8)	1,193 (1.0)	0.7	1.72	1.23 (1.14–1.32)	< 0.001
Parasomnia	82 (0.07)	47 (0.04)	0.03	1.75	1.24 (0.87–1.78)	0.234
Narcolepsy/Cataplexy	41 (0.03)	34 (0.03)	0	1.21	0.87 (0.55–1.38)	0.561
Disturbed Sleep	2,375 (2.1)	1,730 (1.5)	0.6	1.40	0.99 (0.93–1.05)	0.750
Restless Legs	399 (0.3)	252 (0.2)	0.1	1.59	1.12 (0.96–1.31)	0.157
Iron Deficiency	4,489 (4.1)	3,055 (2.6)	1.5	1.55	1.11 (1.06–1.16)	< 0.001
Parkinsonism	10 (0.01)	11 (0.01)	0	0.91	0.26 (0.08–0.80)	0.012

## Discussion

This study demonstrated a significant association between GLP-1RA use and the development of hypersomnolence over both short- and long-term follow-up periods. These findings are consistent with an emerging body of literature suggesting that GLP-1RAs, influence central nervous system pathways involved in sleep regulation. GLP-1 receptors are expressed in the lateral hypothalamus and brainstem nuclei, areas that regulate arousal, REM/non-REM transitions, and circadian rhythms [2–4, 10, 11]. The lateral hypothalamus contains orexin-producing neurons that maintain wakefulness; disruption of this system is a known mechanism in narcolepsy. It is therefore plausible that exogenous GLP-1RA exposure may interfere with orexinergic tone, destabilizing sleep-wake homeostasis, and leading to hypersomnolence and, as observed in our study [11].

Supporting this hypothesis, Khan and Doty reported that GLP-1RAs impair taste function via central pathways, providing indirect evidence of their influence on neurological signaling [12]. Additionally, a system-based review by Fadel et al. emphasized that GLP-1RAs may exert broader neurocognitive and sleep-related effects that remain underexplored [13]. Patient-reported data also reinforce this profile as shown in a systematic review of GLP-1RA experiences. Ibsen et al. documented frequent complaints of fatigue, drowsiness, and poor sleep quality symptoms often underrecognized in clinical settings [14].

While earlier studies have primarily focused on gastrointestinal or metabolic safety profiles such as pancreatitis risk [15] or delayed gastric emptying with aspiration [16] the potential neurologic and sleep-related adverse effects remain poorly characterized. Aldhaleei et al., for instance, found increased gastrointestinal (GI) adverse events in GLP-1RA users, which could contribute to nocturnal reflux and sleep disruption [17]. These findings collectively suggest that GLP-1RAs may influence multiple pathways relevant to sleep, including central neurochemical balance and peripheral GI function.

Although the HR for hypersomnolence indicates a 21–23% relative increase in hazard among the GLP-1RA cohort, the absolute risk was modest. At 1-year the cumulative incidence difference was ~ 0.4%, and at 5-years was ~ 0.7%. This distinction is clinically relevant, as the RR measures may overstate perceived impact when the baseline events are low. Accordingly, the observed association should be interpreted in the context of a small absolute increase in risk, particularly when weighted against the established metabolic and cardiovascular benefits of GLP-1RA therapy.

Overall, our findings add to a growing recognition of the neurophysiological consequences of GLP-1RA therapy. The potential for these agents to disrupt sleep-wake stability through modulation of orexin signaling and REM architecture warrants further mechanistic investigation in future studies.

In our analysis, we also found that GLP-1RA use was significantly associated with increased incidence of iron deficiency at the 5-year endpoint. Prior studies corroborate this relationship, potentially through altered GI absorption or inflammatory modulation [18–20]. Specifically, the mechanistic links between iron deficiency and both reduced absorption and chronic inflammation are often related through the regulatory hormone hepcidin [21]. While obesity can reduce iron absorption through increased hepcidin concentrations [22]. Given the known link between iron deficiency and RLS, this may represent a key pathophysiologic pathway contributing towards the elevated RLS risk observed in our study. RLS is known to contribute to disturbed sleep and affects nearly all patients who are seeking treatment [23, 24]. Therefore, GLP-1RA induced iron deficiency may represent a mechanistic pathway through which these agents contribute to RLS and a downstream sleep disruption.

Our study carries important clinical significance given the limited understanding of sleep related consequences from GLP-1RA use. As these agents gain traction not only in individuals with metabolic disease but also in otherwise healthy populations, their clinical reach continues to expand. Concurrently, as they are increasingly explored for neurologic indications, recognition of potential adverse effects such as somnolence, parasomnia, and sleep fragmentation becomes essential [25, 26]. These findings may aid clinicians to anticipate and manage these side effects earlier, especially in patients with baseline hypersomnia or sleep related disorders. Additionally, this knowledge can help to guide more personalized and informed risk benefit assessments when considering GLP-1RA therapy in clinical practice.

Though our results are statistically and clinically significant, there are several limitations to this study that must be acknowledged. First, the study relied on de-identified administrative data which precluded access to patient level data such as symptom severity, medication adherence, or objective sleep architecture metrics from polysomnography.

Secondly, given the retrospective cohort design, causal inference cannot be established despite rigorous propensity score matching and time to event analysis. Unmeasured confounding variables such as socioeconomic status, environmental sleep conditions, or unrecorded psychiatric comorbidities may have influenced the observed associations. There is also the consideration of diagnostic coding errors or omission that could have introduced misclassification bias. As with all retrospective observational studies, the possibility of confounding by indication or other systemic differences between groups cannot be fully excluded. This analysis also did not differentiate between specific GLP1-RA agents, dosages, duration of treatment, or adherence patterns. The observed association between GLP-1RA use and iron deficiency also raises the possibility of mediation effects. Iron deficiency is a known contributor to RLS and sleep fragmentation. Hence, some sleep related findings may be due to indirect pathways rather than CNS effects of GLP-1RA therapy. Our study also used polysomnography as an inclusion criterion which likely selected a population already undergoing evaluation for sleep disturbances limiting generalizability.

However, this study has several strengths. To our knowledge, this is the first large scale study evaluating GLP-1RA in relation to hypersomnolence and related outcomes using real world data. We utilized a large, multicenter population with diverse demographics which enhances generalizability. Our study also reduced baseline bias via propensity score matching, and time to event analysis provided insight to early and delayed risks.

## Conclusion

In conclusion, our study found that GLP-1RA use was associated with a statistically significant increase in hazard of hypersomnolence at both 1- and 5-year endpoints. Although the relative increase in hazard was consistent, the absolute risk increase was modest, remaining below 1% over 5 years. An increased hazard of iron deficiency at 5-years and decreased hazard of Parkinsonism at 5-years were observed, though the latter finding was based on low even counts and should be interpreted with caution. Future research should include prospective studies with polysomnographic data, pharmacodynamic exploration of CNS effects, and stratification by psychiatric and neurologic comorbidities. As the role of GLP-1RA therapy continues to expand, it is critical to understand the broader impact on sleep, cognition, and underlying mechanisms of these associations.

## Supplementary Information

Below is the link to the electronic supplementary material.

Supplementary Material 1

## Data Availability

The data that support the findings of this study were obtained from the TriNetX Research Network, a federated, multi-institutional clinical research platform that provides access to de-identified electronic health record data. Due to data use agreements and patient privacy protections, these data are not publicly available and cannot be shared directly by the authors. Researchers with appropriate institutional approvals may access the data through the TriNetX platform (https://www.trinetx.com) subject to TriNetX policies and regulatory requirements.

## Abbreviations

CI: Confidence interval
CNS: Central nervous system
CPT: Current Procedural Terminology
GI: Gastrointestinal
GLP-1RA: Glucagon-like peptide-1 receptor agonist
HCO: Healthcare organization
HIPAA: Health Insurance Portability and Accountability Act
HR: Hazard ratio
ICD-10-CM: International Classification of Diseases, Tenth Revision, Clinical Modification
LOINC: Logical Observation Identifiers Names and Codes
REM: Rapid eye movement
RLS: Restless legs syndrome
RR: Risk ratio
STROBE: Strengthening the Reporting of Observational Studies in Epidemiology
T2DM: Type 2 diabetes mellitus
TNX: TriNetX curated code
