# Supplementary material for: Association of GLP-1 Receptor Agonist Use with Hypersomnolence: A Real-world Cohort Analysis
Source: J Diabetes Metab Disord. 2026 Mar 18;25(1):117. doi: 10.1007/s40200-026-01929-0 (PMC13000040; doi:10.1007/s40200-026-01929-0)
Supplement: Supplementary file 1 — Supplementary Material 1 [file 40200_2026_1929_MOESM1_ESM.docx]

STROBE Statement—checklist of items that should be included in reports of observational studies

|  | Item No. | Recommendation | Page  No. | Relevant text from manuscript |
| --- | --- | --- | --- | --- |
| **Title and abstract** | 1 | (*a*) Indicate the study’s design with a commonly used term in the title or the abstract | Title p.2; | “Association of GLP-1 Receptor Agonist Use with Hypersomnolence: A Real-World Cohort Analysis” … |
|  |  | (*b*) Provide in the abstract an informative and balanced summary of what was done and what was found | Abstract p.4 | “retrospective cohort study… propensity matched…” |
| Introduction | | | |  |
| Background/rationale | 2 | Explain the scientific background and rationale for the investigation being reported | Introduction p.6 | “Accumulating evidence suggests GLP-1RAs exert significant CNS effects… potentially leading to hypersomnolence… sleep-related disturbances.” |
| Objectives | 3 | State specific objectives, including any prespecified hypotheses | Introduction p.6 | “This study aims to investigate the association between GLP-1RA use and hypersomnolence, parasomnia, and related outcomes…” |
| Methods | | | |  |
| Study design | 4 | Present key elements of study design early in the paper | Abstract p.4; Methods p.7 | “A retrospective cohort study was done using TriNetX’s research network…” |
| Setting | 5 | Describe the setting, locations, and relevant dates, including periods of recruitment, exposure, follow-up, and data collection | Methods – Study Design p.7 | “TriNetX… ~157 million patients from 107 HCOs… July 2025.” |
| Participants | 6 | (*a*) *Cohort study*—Give the eligibility criteria, and the sources and methods of selection of participants. Describe methods of follow-up | Methods – Cohorts p.7–8 | “Patients aged 18–50 with T2DM or obesity… excluded prior antidepressant use… matched 1:1.” |
|  |  | (*b*) *Cohort study*—For matched studies, give matching criteria and number of exposed and unexposed | Methods – Cohorts p.7–8 | “Patients aged 18–50 with T2DM or obesity… excluded prior antidepressant use… matched 1:1.” |
| Variables | 7 | Clearly define all outcomes, exposures, predictors, potential confounders, and effect modifiers. Give diagnostic criteria, if applicable | Methods – Outcomes p.8 | “Primary outcome… hypersomnolence (G47.1, R40.0)… secondary outcomes… parasomnia, RLS, iron deficiency, etc.” |
| Data sources/ measurement | 8* | For each variable of interest, give sources of data and details of methods of assessment (measurement). Describe comparability of assessment methods if there is more than one group | Methods – Study Design & Outcomes p.7–8 | “Diagnoses: ICD-10-CM; labs: LOINC; procedures: CPT; medications: TNX codes.” |
| Bias | 9 | Describe any efforts to address potential sources of bias | Methods – Cohorts & Statistical Analysis p.7–8 | “Excluded antidepressant use… propensity matched for demographics, comorbidities, medications… SMD <0.10.” |
| Study size | 10 | Explain how the study size was arrived at | Methods – Cohorts p.7–8; Results p.9 | “172,187 GLP-1 users, 2,577,308 controls… matched cohorts of 118,456 patients.” |

Continued on next page

| Quantitative variables | 11 | Explain how quantitative variables were handled in the analyses. If applicable, describe which groupings were chosen and why | Statistical Analysis p.8 | “Continuous variables as mean ± SD; categorical as counts and percentages.” |
| --- | --- | --- | --- | --- |
| Statistical methods | 12 | (*a*) Describe all statistical methods, including those used to control for confounding | Methods – Cohorts & Statistical Analysis p.7–8 | “propensity matched for demographics, comorbidities, medications… SMD <0.10.” |
|  |  | (*b*) Describe any methods used to examine subgroups and interactions |  | N/A |
|  |  | (*c*) Explain how missing data were addressed |  | N/A |
|  |  | (*d*) *Cohort study*—If applicable, explain how loss to follow-up was addressed |  | N/A |
|  |  | (*e*) Describe any sensitivity analyses |  | N/A |
| Results | | | | |
| Participants | 13* | (a) Report numbers of individuals at each stage of study—eg numbers potentially eligible, examined for eligibility, confirmed eligible, included in the study, completing follow-up, and analysed | Results p.9–10; Tables 1–3 p.16–19 | “After inclusion/exclusion… matched cohorts of 118,456… excluded prior outcome history per outcome.” |
|  |  | (b) Give reasons for non-participation at each stage | Methods – Cohorts & Statistical Analysis p.7–8 | “Excluded antidepressant use… propensity matched for demographics, comorbidities, medications… SMD <0.10.” |
|  |  | (c) Consider use of a flow diagram |  |  |
| Descriptive data | 14* | (a) Give characteristics of study participants (eg demographic, clinical, social) and information on exposures and potential confounders | Results p.9; Table 1 p.16–17 | “Baseline characteristics well balanced… mean age ~39… ~62% female.” |
|  |  | (b) Indicate number of participants with missing data for each variable of interest |  | N/A |
|  |  | (c) *Cohort study*—Summarise follow-up time (eg, average and total amount) | Results p.9 Tables 2-3 | “At both the 1 year and 5-year endpoints” |
| Outcome data | 15* | *Cohort study*—Report numbers of outcome events or summary measures over time | Results p.9–10; Tables 2–3 p.18–19 | “Hypersomnolence RR 1.61 at 1 yr, 1.72 at 5 yr… other significant outcomes included…” |
| Main results | 16 | (*a*) Give unadjusted estimates and, if applicable, confounder-adjusted estimates and their precision (eg, 95% confidence interval). Make clear which confounders were adjusted for and why they were included | Results p.9–10; Tables 2–3 p.18–19 | “Propensity-matched estimates… RR, HR, CI reported for each outcome.” |
|  |  | (*b*) Report category boundaries when continuous variables were categorized |  | N/A |
|  |  | (*c*) If relevant, consider translating estimates of relative risk into absolute risk for a meaningful time period |  | N/A |

Continued on next page

| Other analyses | 17 | Report other analyses done—eg analyses of subgroups and interactions, and sensitivity analyses |  | N/A |
| --- | --- | --- | --- | --- |
| Discussion | | | | |
| Key results | 18 | Summarise key results with reference to study objectives | Discussion p.10–11 | “GLP-1RA use significantly associated with hypersomnolence and parasomnia at both timepoints…” |
| Limitations | 19 | Discuss limitations of the study, taking into account sources of potential bias or imprecision. Discuss both direction and magnitude of any potential bias | Discussion p.12 | \|  \| \| --- \| \| “Relied on de-identified data… cannot establish causality… possible coding errors.” \| \| |
| Interpretation | 20 | Give a cautious overall interpretation of results considering objectives, limitations, multiplicity of analyses, results from similar studies, and other relevant evidence | Discussion p.10–13 | “Findings consistent with mechanistic data on orexin modulation… potential role in RLS via iron deficiency.” |
| Generalisability | 21 | Discuss the generalisability (external validity) of the study results | \|  \| \| --- \|  \| Discussion p.13 \| \| --- \| | “Large, multicenter population with diverse demographics enhances generalizability.” |
| Other information | |  | | |
| Funding | 22 | Give the source of funding and the role of the funders for the present study and, if applicable, for the original study on which the present article is based | Funding p.13 | “No specific grant funding… no funder role.” |

*Give information separately for cases and controls in case-control studies and, if applicable, for exposed and unexposed groups in cohort and cross-sectional studies.

**Note:** An Explanation and Elaboration article discusses each checklist item and gives methodological background and published examples of transparent reporting. The STROBE checklist is best used in conjunction with this article (freely available on the Web sites of PLoS Medicine at http://www.plosmedicine.org/, Annals of Internal Medicine at http://www.annals.org/, and Epidemiology at http://www.epidem.com/). Information on the STROBE Initiative is available at www.strobe-statement.org.
